# A mixed method study exploring adherence to and acceptability of small quantity lipid-based nutrient supplements (SQ-LNS) among pregnant and lactating women in Ghana and Malawi

**DOI:** 10.1186/s12884-016-1039-0

**Published:** 2016-08-30

**Authors:** Moses K. Klevor, Seth Adu-Afarwuah, Per Ashorn, Mary Arimond, Kathryn G. Dewey, Anna Lartey, Kenneth Maleta, Nozgechi Phiri, Juha Pyykkö, Mamane Zeilani, Ulla Ashorn

**Affiliations:** 1 Program in International and Community Nutrition, Department of Nutrition, University of California, Davis, Davis, CA USA; 2 Department of Nutrition and Food Science, University of Ghana, Legon, Ghana; 3 Center for Child Health Research and Department of Paediatrics, University of Tampere School of Medicine and Tampere University Hospital, Tampere, Finland; 4 School of Public Health and Family Medicine, University of Malawi College of Medicine, Blantyre, Malawi; 5 Department for International Health, University of Tampere School of Medicine, Tampere, Finland; 6 Nutriset S.A.S., Malaunay, France

**Keywords:** Small-quantity lipid-based nutrient supplements, Adherence, Acceptability, Women, Pregnancy, Lactation, Ghana, Malawi

## Abstract

**Background:**

Supplementing pregnant and lactating mothers with small quantity lipid-based nutrient supplements (SQ-LNS) has resulted in improvements in birth outcomes in some low-income settings. In order to be effective, SQ-LNS must be consumed regularly over sustained periods.

**Methods:**

The objective was to assess and compare acceptability of and adherence to SQ-LNS consumption among pregnant and lactating women in Ghana and Malawi throughout 12 months of supplementation. We enrolled women before 20 gestation weeks into randomized trials in Ghana (*n* = 1320) and Malawi (*n* = 869). In the SQ-LNS group participants received a 20 g sachet of supplement per day during pregnancy and the first 6 months of lactation. In the control groups participants received multiple micronutrients (MMN) during pregnancy and lactation or iron and folic acid (IFA) during pregnancy and calcium during lactation. We used questionnaires to collect data on self-reported adherence to daily use of supplements and conducted in-depth interviews with women in the SQ-LNS group to examine acceptability.

**Results:**

The mean self-reported adherence during the supplementation period was lower in Ghana (79.9 %) than in Malawi (91.7 %) for all supplements (difference 11.8 %, *P* < 0.001). Over time, adherence increased in Malawi but decreased in Ghana. In both countries, adherence in the SQ-LNS group was non-inferior to that in the control groups. Participants typically reported consuming SQ-LNS as instructed but when interviewers queried about experiences, most of the women described incidents of non-adherence. A usual reason for not consuming SQ-LNS was nausea and vomiting during pregnancy. Especially in Malawi, women reported sharing SQ-LNS with families and friends. Sustained use of SQ-LNS was attributed to expected health benefits and favorable sensory attributes. Often women compared their pregnancy to previous ones, and were of the view that SQ-LNS made a positive difference.

**Conclusion:**

Self-reported sustained adherence to consume SQ-LNS daily was high in both sites but lower in Ghana than in Malawi. In Ghana, adherence decreased over time whereas in Malawi adherence increased. Acceptability and adherence appeared interlinked, complex and context-related. Sustained consumption of SQ-LNS may require tailoring interventions by context.

**Trial registration:**

The Ghana trial was registered at clinicaltrials.gov as NCT00970866, and the Malawi trial as NCT01239693.

**Electronic supplementary material:**

The online version of this article (doi:10.1186/s12884-016-1039-0) contains supplementary material, which is available to authorized users.

## Background

Linear growth failure in childhood, stunting, is the most common growth deficit globally. Stunting prevalence has been decreasing slowly and 165 million children were estimated to be stunted in 2011 [10]. Typically, stunting begins in utero and continues especially during the vulnerable period of complementary feeding [25, 27]. Among other things, inadequate maternal diet during pregnancy and lactation, combined with increased nutrient requirements, play a crucial role in the etiology of stunting [1]. In the 2030 Agenda for Sustainable Development, reduction of stunting is used as an indicator for the achievement of the nutrition target (http://www.un.org/sustainabledevelopment/hunger). As more global attention is directed towards length gain in infancy and childhood, there is a need to better understand the potential of interventions towards reaching the global goal.

Small-quantity lipid-based nutrient supplements (SQ-LNS) are designed to be used as one component in nutrition interventions for pregnant and lactating women and children from 6 months onwards to prevent moderate malnutrition leading to stunting. SQ-LNS are a family of energy-dense pastes that contain protein, carbohydrates, and micronutrients embedded in a lipid base, to be mixed with regular foods [7]. There have been several trials on supplementing children with SQ-LNS [2, 8, 9, 21, 26]. Supplementing pregnant and lactating mothers with SQ-LNS has resulted in improvements in birth outcomes in some low-income settings [4, 8, 9, 24]. Both maternal and child supplementation have produced mixed efficacy results with respect to child growth; supplementing women with SQ-LNS during pregnancy and the first 6 months of lactation and their children between 6 and 18 months of age did not impact child length at 18 months in Malawi [9], but had a positive impact on child length at 18 months in Ghana [5]. Differences in the study contexts and participant adherence to the intervention may possibly explain this heterogeneity [22].

Among others factors, the efficacy results of a complementary feeding intervention depend on sustained consumption of the products, i.e. adherence. In the present report we analyze longitudinal adherence among participants who were receiving SQ-LNS during pregnancy and the first six months of lactation as part of two randomized trials in Ghana and in Malawi. The objectives of the present study were twofold. Firstly, using quantitative data, we examined self-reported adherence to consume SQ-LNS and the control products (multiple micronutrients tablets, MMN and iron and folic acid tablet, IFA) daily. We compared mean adherence between the two countries and between SQ-LNS and control groups. Secondly, using qualitative data, we assessed participants’ experiences and acceptability of SQ-LNS.

## Methods

### Trial context

We collected the data as part of iLiNS-DYAD trials in Ghana and in Malawi and the main results have been published earlier [4, 8, 9]. In brief, the main aim was to test a hypothesis that provision of 20 g sachet SQ-LNS to mothers during pregnancy and during the first 6 months of lactation and to their infants from 6 to 18 months of age would result in favorable birth outcomes (birth length and weight) and improved child growth compared to children of mothers in the two control groups: one receiving either multiple micronutrient tablets (MMN) during pregnancy and lactation or iron and folic acid tablets during pregnancy (IFA) and placebo tablets during lactation. The SQ-LNS were formulated to provide 1x or 2x the recommended dietary allowance (RDA) of the chosen nutrients both for the mothers and the children [7]. We have already published trial settings and the growth results: In Ghana there was an increase in birth size of infants of primiparous women who received SQ-LNS and the growth effect was sustained till 18 months (iLiNS project, [5]). In Malawi there was no effect on growth at birth or at 18 months [8, 9].

This paper reports on acceptability of SQ-LNS and on adherence to recommended consumption, relative to instructions given by research nurses to each woman at enrolment: 1) she should take the supplement every day while in study until 6 months during lactation, 2) she should take the supplement without sharing it, 3) she should mix SQ-LNS with a small amount of food before eating the rest of the meal. This was to ensure that the entire content of each SQ-LNS sachet was consumed.

### Study areas and participants

In Ghana, the trial was conducted at the Somanya-Kpong area, about 70 km north of Accra (national capital). Participants were enrolled from the four main health facilities in the area. The study area consisted of several adjoining, semi-urban communities stretching about 20 km. The area was served by electricity and potable water from the public supply. Most inhabitants were subsistence farmers or petty traders and were neither poor nor rich by Ghanaian standards. The staple diet consisted of maize, cassava, rice, fish, and leafy vegetables [4].

In Malawi, the trial was conducted in Mangochi District situated at the southern end of Lake Malawi. Participants were enrolled at antenatal clinics in a government-run hospital and health centers and in a private hospital. The catchment area was predominantly rural with no electricity supply and water drawn from wells and boreholes. Most inhabitants subsisted on farming maize and fishing. The staple diet consists of stiff maize porridge consumed with sauce made of vegetables and some fish or meat. Households often suffered from lack of food and seasonal hunger before the new harvest [8, 9].

The target population comprised pregnant women who came for antenatal care at any of the study hospitals or clinics. The study teams screened and enrolled participants who met the inclusion criteria: ultrasound scan-confirmed pregnancy of no more than 20 completed gestation weeks, having antenatal cards with complete medical and examination history (Ghana), residence in the defined catchment area, availability during the period of the study, and signed or thumb-printed informed consent. Exclusion criteria were: age less than 15 years for Malawi and less than 18 y for Ghana, antenatal cards indicating HIV infection status (Ghana), need for frequent medical attention due to a chronic health condition, diagnosed asthma treated with regular medication, severe illness warranting hospital referral, history of peanut allergy, history of anaphylaxis or serious allergic reaction to any substance requiring emergency medical care, pregnancy complications evident at enrolment visit, earlier participation in the iLiNS-DYAD trial (during a previous pregnancy), or concurrent participation in any other clinical trial.

### Data collection

We used mixed methods to collect data in order to provide a comprehensive analysis of adherence to and acceptability of SQ-LNS [11]. For quantitative analysis the sample size was determined by the main studies [4, 9]. In Malawi, research assistants visited the homes of the participants weekly. They delivered a two-wk ration of SQ-LNS, IFA or MMN at every other visit. Every week, they administered a questionnaire about adherence to recommended daily consumption of the supplement during the previous week. In Ghana, home visit teams went to the women biweekly to deliver a two-week supply of supplements and documented adherence to the recommended daily consumption of the supplements using a questionnaire-based interview.

For qualitative analysis, female researchers who had not previously interacted with the study participants made two home visits to a subset of mothers who received SQ-LNS and conducted in-depth interviews about their experiences with the supplements using a thematic interview guide. The first interview was done soon after enrolment in early pregnancy and the second interview between 6 and 12 months postpartum. We conducted about 30 interviews which allowed us to select the interviewees purposively to include mothers of different ages, educational levels, parity and living situations. In Ghana, where different languages were spoken in the study area (Twi, Ewe, Ga, Krobo), the researcher used a thematic interview guide in English but conducted the interviews using vernacular. These female researchers were trained to pose the interview questions in a consistent manner. In Malawi, we translated the thematic guides into the local languages (Chichewa, Chiyao). In both settings, an interpreter fluent in the local language was present if the researcher was not fluent in the language used for the interview.

### Data analysis

**Fig. 1:**
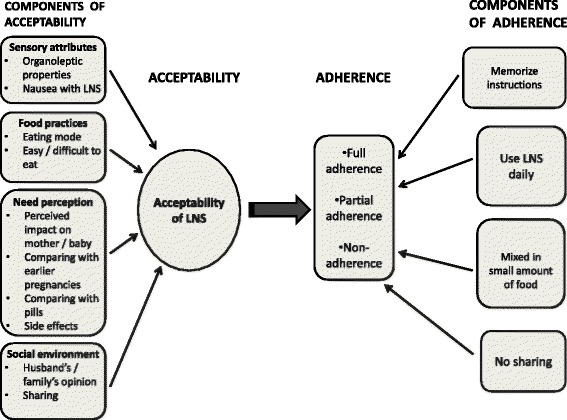
Concept map of adherence and acceptability

Figure 1 presents a concept map for data analysis which we created based on previous literature and preliminary examination of data. Our assumption was that the effectiveness of the intervention depends on how meticulously women followed the instructions to consume the supplements daily; in other words, adherence. A necessary interconnected concept is acceptability which has been conceptualized as a favorable attitude towards a product, predisposing a person to be willing to use it according to instructions [29].

For the present analysis, we considered adherence as an etic (outsider) perspective constructed by the trial designers. For capturing this perspective, we analyzed the aspects of data covering the instructions the team gave and whether products were reported to be used as intended. We considered acceptability as an emic (insider) perspective and we analyzed the segments of transcripts where women narrated how they had used SQ-LNS and justification for their behavior.

In Malawi, data clerks entered questionnaire-based weekly data into a tailor-made database through scanning, digital character recognition, and manual verification of critical variables and suspicious entries. In Ghana, data clerks double-entered questionnaire-based biweekly data into a computer database manually, with regular quality control checks by the data quality control officer. We analysed these data for biweekly and mean adherence percentages based on self-reported intake of the products (i.e. days consumed) from enrolment till 25 weeks during lactation or end of participation, whichever came first. We based the analysis on the principle of modified intention-to-treat, i.e. we included all randomized participants into the analysis if they had 4 or more visits during pregnancy and 4 or more visits during lactation.

Our preliminary analysis of in-depth interviews suggested lower adherence in Ghana than in Malawi. For testing a hypothesis of adherence being lower among Ghanaian than among Malawian women, for all supplement types, we performed a Mann–Whitney U test using the overall adherence percentage. To test if adherence was lower for SQ-LNS (a novel supplement) than for IFA and MMN (controls), we predefined a 10 % non-inferiority margin sufficient for practical relevance. For testing the hypothesis of an increase in adherence percentage over time, we calculated the rate of change in the biweekly adherence percentage within each study group. We created a regression model and calculated a slope for adherence percentage over time for each participant. For comparing adherence between pregnancy and lactation within countries we performed a Wilcoxon signed-rank test. For all the quantitative analysis, we used data collected from 20 weeks gestation to 25 weeks after delivery. However, visits occurring after 40 weeks gestation were excluded, since there were very few. Additionally, we excluded the first wk after delivery when calculating adherence during lactation, because data collected 1 week post-partum may have included a wk of pregnancy and a wk of lactation.

Researchers recorded the in-depth interviews and transcribed them verbatim into the language used during the interview, and then translated into English. For organising and analysing the data, we used a modified framework method, which belongs to the family of content analysis and is geared towards policy and practice-oriented findings [17]. According to this method, we first familiarized ourselves with the interviews; based on that, we agreed on the codes to be used in an initial analytical framework and formed a matrix which was used for interpreting the data [14, 17]. A distinctive aspect of framework analysis is that it allows themes to develop both from the research questions and from the narratives of research participants [28]. Themes were developed in relation to participants’ experiences with the use of SQ-LNS, including adherence accounts, sensory attributes and side effects, food preferences and convenience, needs perception and benefits, and the social environment. In order to guard against researcher bias and help ensure that the data accurately reflected the perception of the interviewees, two researchers (MKK, UA) generated codes and themes independently. The coded segments of the transcripts were shared and compared for discrepancies. In very rare instances when there was disagreement, both researchers discussed the codes and themes and arrived at a consensus. The two researchers then produced a thematic framework together; the framework was revised throughout the coding process to ensure it was appropriate for the data, and then applied systematically to both coded and yet-to-be coded transcripts. Additionally, we used focused coding, concentrating on interview segments that were about women’s experiences with consuming SQ-LNS [13]. Using this approach, we summarized data into thematic matrices for identifying social, cultural and behavioral explanations for consuming or not consuming SQ-LNS on a daily basis. We used Atlas.ti software, version 7 for coding.

## Results

### Success of follow-up

We collected data in Ghana from December 2009 through August 2012 and in Malawi from February 2011 to August 2012. In Ghana, 1320 and in Malawi 869 pregnant women were enrolled into the study. Background characteristics of the participants at enrollment are presented in Table 1. Participant flow and reasons for loss to follow-up are presented in Additional file 1: Figure S1. In Ghana, 127 (9.6 %) and in Malawi 150 (17.3 %) women were lost to follow-up during pregnancy and the first 6 months during lactation.

**Table 1:** Participant and household characteristics at enrolment, by intervention group

Characteristic	Ghana				Malawi			
	SQ-LNS	MMN	IFA	P-value^*^	SQ-LNS	MMN	IFA	P-value^*^
Number of participants	440	439	441		288	291	290	
Age y, mean (SD)	26.9 (5.6)	26.9 (5.4)	26.4 (5.6)	0.24	25.1 (6.3)	24.5 (5.8)	25.0 (6.0)	0.50
Schooling y, mean (SD)	7.6 (3.9)	7.6 (3.6)	7.8 (3.5)	0.60	4.0 (3.7)	4.0 (3.4)	3.8 (3.5)	0.79
Proportion of married	92.1 %	93.6 %	92.5 %	0.65	85.6 %	85.6 %	84.8 %	0.95
Proportion of primiparous women	33.4 %	32.6 %	35.4 %	0.67	24.0 %	23.4 %	23.2 %	0.97
Household asset index, mean (SD)^a^	−0.09 (1.00)	0.06 (0.97)	0.04 (1.02)	0.05	0.15 (1.94)	0.00 (1.78)	−0.03 (1.85)	0.47
Head of household is respondent’s husband	67.0 %	64.7 %	65.0 %	0.72	45.5 %	43.6 %	44.1 %	0.91

^*^Chi-squared test or ANOVA/Kruskal-Wallis

^a^Standardized within each site to a mean of 0 and SD of 1. Comparison across sites is not meaningful

We had transcripts of interviews with 32 women in Ghana and 30 women in Malawi during early pregnancy. During lactation we re-interviewed 30 women in Ghana and 27 women in Malawi. In Malawi, three of the infants died before the second interview, with one being a stillbirth. In Ghana, one infant died and one mother ended participation in the study. We did not attempt to interview these women.

### Self-reported adherence to consume supplement daily

**Fig. 2:**
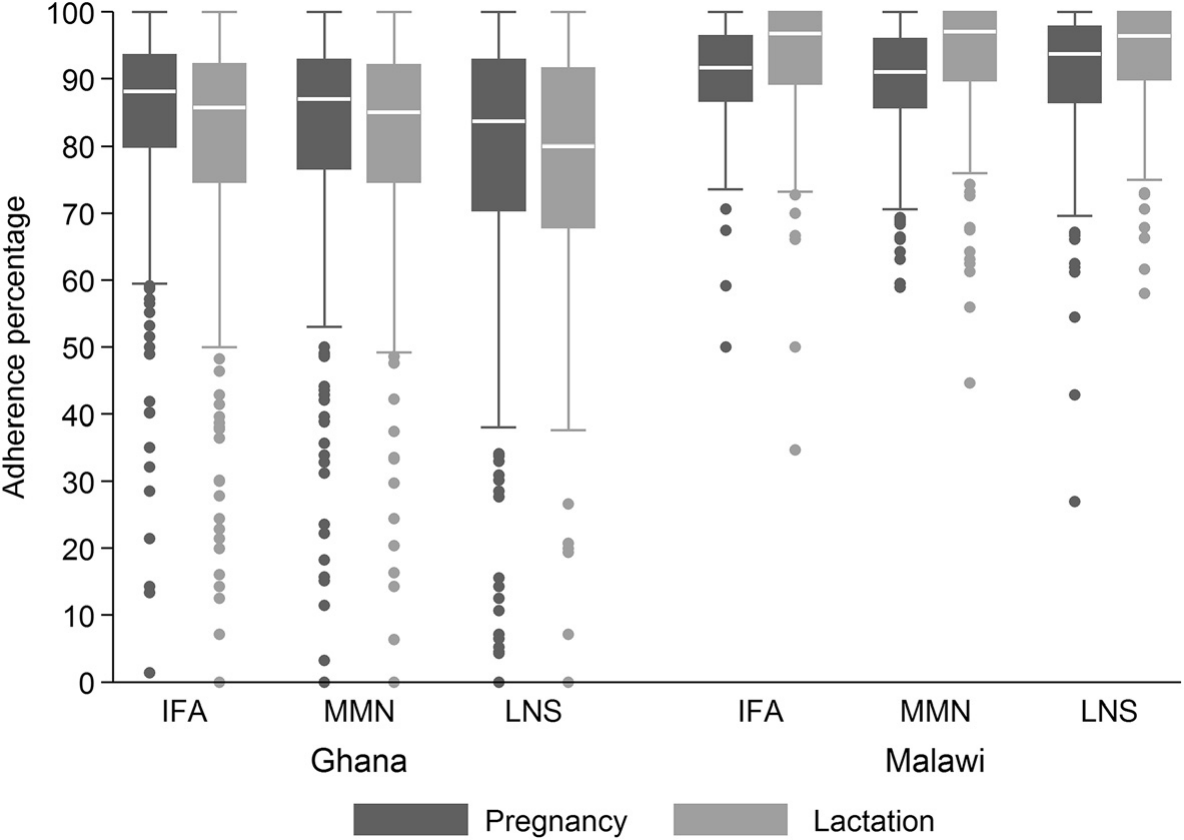
Overall adherence in pregnancy and lactation, by country and supplement. Median (N) in Ghana for pregnancy/lactation 88.1 %/85.7 % (399), 87.0 %/85.0 % (403) and 83.7 %/80.0 % (391) for IFA, MMN and SQ-LNS, respectively. Median (N) in Malawi for pregnancy/lactation 91.7 %/96.7 % (241), 91.0 %/97.0 % (240) and 93.8 %/96.4 % (238) for IFA, MMN and SQ-LNS, respectively

The mean (SD) self-reported overall adherence to supplement consumption was 79.9 % (16.9 %) in Ghana and 91.7 % (6.8 %) in Malawi (Mann–Whitney *U* test for equality of percentages: z = −20.48, *P* < 0.001). In both countries, some participants reported close to full adherence but some reported that they consumed the supplements very seldom (Fig. 2). The mean overall adherence was 9–16 %-points lower in Ghana than in Malawi for each supplement type, both during pregnancy and lactation (*P* < 0.001), Table 2.

**Table 2:** Self-reported overall adherence in Ghana and in Malawi during pregnancy and lactation, by intervention group

Pregnancy	Ghana	Malawi	Difference in means (95 % CI^a^)	P-value^*^
Total, % (n)	81.5 (1193)	90.1 (719)	−8.5 (−9.9, −7.1)	<0.001
IFA, % (n)	84.5 (399)	90.5 (241)	−6.0 (−7.9, −4.0)	<0.001
MMN, % (n)	81.9 (403)	89.2 (240)	−7.3 (−9.5, −5.0)	<0.001
SQ-LNS, % (n)	78.2 (391)	90.6 (238)	−12.4 (−15.3, −9.6)	<0.001
Lactation	Ghana	Malawi	Difference in means (95 % CI^a^)	P-value^*^
Total, % (n)	78.5 (1193)	93.4 (719)	−14.9 (−16.5, −13.3)	<0.001
IFA, % (n)	80.4 (399)	93.3 (241)	−12.9 (−15.4, −10.4)	<0.001
MMN, % (n)	79.9 (403)	93.4 (240)	−13.5 (−16.1, −10.8)	<0.001
SQ-LNS, % (n)	75.0 (391)	93.5 (238)	−18.5 (−21.6, −15.4)	<0.001

^*^Pairwise comparisons of means using Wilcoxon-Mann-Whitney test

^a^Confidence interval obtained from t-test

**Fig. 3:**
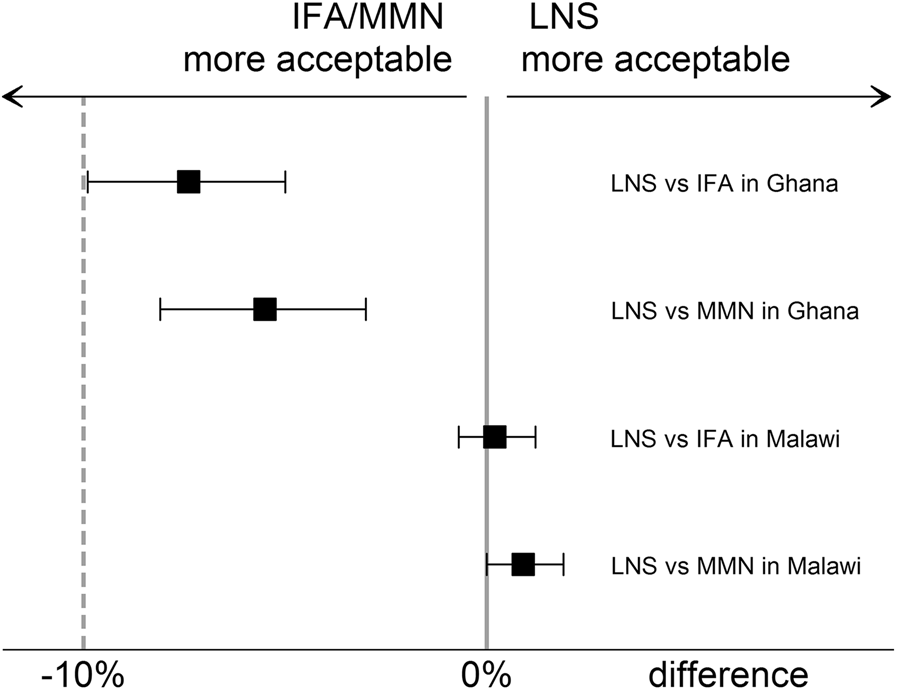
Non-inferiority analysis, by country and supplement. In Ghana mean (90 % of the mean) for adherence for IFA and MMN was 82.4 % (74.2 %) and 80.8 % (72.7 %), respectively, and mean (95 % CI) for adherence for SQ-LNS was 76.3 % (74.3 %, 78.3 %). In Malawi mean (90 % of the mean) for adherence for IFA and MMN was 91.8 % (82.6 %) and 91.2 % (82.1 %), respectively, and mean (95 % CI) for adherence for SQ-LNS was 92.0 % (91.1 %, 92.9 %). Figure values converted to present difference. Center of box indicates the mean and wings represent the 95 % CI

Comparison between the supplemented groups indicated non-inferiority of the adherence in the SQ-LNS group as compared to the IFA and MMN groups in Malawi and Ghana (lower bound of 95 % CI above 90 % of mean of IFA and MMN) (Fig. 3).

**Fig. 4:**
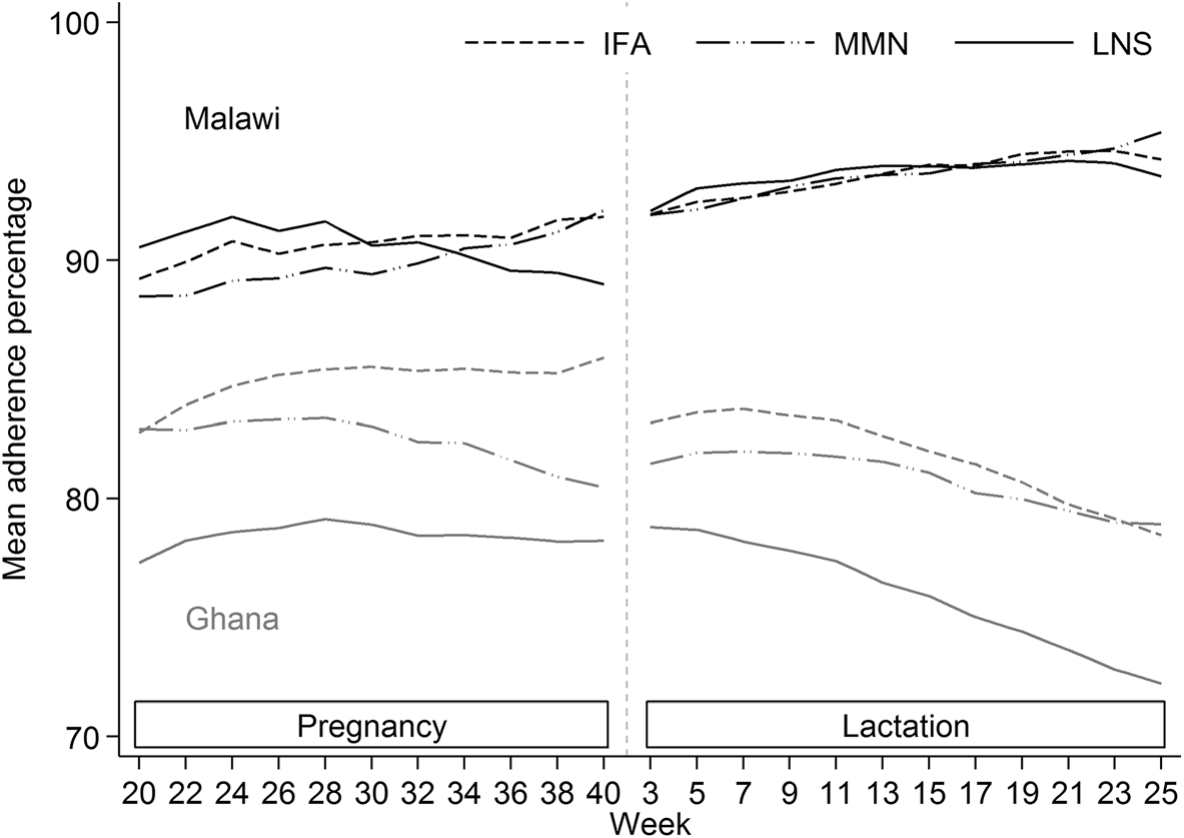
Adherence time trends, by country and intervention product. Lowess curves, no weighting, bandwidth 0.6

The mean overall adherence increased over time in Malawi in all supplemented groups (*P* < 0.001 for each slope, one-sample t-test for rate of change) whereas in Ghana, the mean overall adherence decreased over time in all supplemented groups (*P* < 0.001 for each slope, one-sample t-test for rate of change) (Fig. 4).

In Ghana mean (SD) adherence was 81.5 % (17.6 %) in pregnancy and 78.5 % (20.8 %) during lactation (*P* < 0.001, Wilcoxon signed-rank test). In Malawi mean (SD) adherence was 90.1 % (8.9 %) in pregnancy and 93.4 % (8.6 %) during lactation (*P* < 0.001, Wilcoxon signed-rank test).

### Adherence accounts in in-depth interviews

In-depth interviews offered some insight into the reported weekly adherence to SQ-LNS (referred to as *Nkatepa* in Ghana and as *Chiponde Chatsopano* in Malawi). Almost all women memorized the instructions correctly and initially reported that they had consumed SQ-LNS as instructed. Additionally, they emphasized that the instructions were given by a nurse (some Malawians said doctor) at a health facility and therefore they should be closely obeyed. However, in the course of the interviews, when the interviewer queried about experiences with SQ-LNS using probes, most of the women described incidents of non-adherence. Along the same lines, most of the initial reactions to questions about experiences with SQ-LNS were positive but during the interviews some women also brought forward some negative experiences. Components of acceptability and adherence were different among individuals and also in Ghana as compared to Malawi, and when appropriate the text below reflects this variation.

### Sensory attributes and side effects

In both countries, sensory attributes (taste, smell, palatability, texture, color) were central in interview accounts. In general in Ghana, participants were more critical, discussed organoleptic properties in more detail than in Malawi and expressed various sentiments about the properties and qualities of *Nkatepa*. According to several of the interviewed Ghanaian women, they noticed the smell (scent) or taste of groundnut and did not like it. Others indicated that they felt on their tongues or tasted or smelled the “medicines” that were added to the *Nkatepa*. Some women described it as being too sweet, others described it as being bitter or having a bitter aftertaste; a few others described it as neither sweet nor bitter but tasteless. For some women, the taste or texture of *Nkatepa* either prevented them from enjoying it or made it difficult for them to consume it as described by this interviewee: *Like they said, they grind the blood tonic- the green one, the red and folic acid and mix it with the groundnut. So if you open it and you are taking it you’ll notice that what was grinded and mixed has some particles left in it. So at times… you’ll feel it on your tongue* (Ghana 1706, 1^st^ interview).

In the Malawi interviews, statements were more positive and women typically stated that they liked *Chiponde*, including its organoleptic properties. However, when probed further, some Malawian women also said that *Chiponde* gave a bad aftertaste or was bitter: *It was tasty, mixing it with porridge it was really tasty … but just to eat it like that it felt a bit bitter* (Malawi 4026, 2^nd^ interview). Some reported adding sugar to the *Chiponde* to make it sweeter.

An additional feature women related to organoleptic properties of SQ-LNS was nausea. In both countries, several women indicated that they experienced nausea or vomiting after they consumed SQ-LNS. Some of the women noted that they experienced nausea and vomiting when they mixed the SQ-LNS with some particular foods, but if they mixed it with other foods they did not experience any difficulties. However, it appears that the majority of the nausea and vomiting experienced by the women was due to their physiological state in early pregnancy. When interviewed postpartum, eating had typically become easier, as this interviewee from Malawi who stopped eating *Chiponde* for 2 months said: *When I mixed with porridge, I was vomiting, so I stopped, I wasn’t eating, I started [eating] when I was 7 months [pregnant]* (Malawi 3054, 2^nd^ interview). Some mothers in Ghana also reported similar behavior: *When I was just pregnant when I mixed the medicine with food and ate, sometimes I vomited. But when the pregnancy was growing, when I took it, nothing happened to me* (Ghana 1100, 2^nd^ interview).

However, some women explained how they were unable to eat SQ-LNS even if they tried both during pregnancy and postpartum as stated by one study participant: *I just prepare it for the children and when they are stirring, I shouldn’t be near because when the smell reaches me, I don’t feel good, just the smell*. During the second interview she still gave it to the children: *Yes, even now if I get a whiff of the smell I want to vomit* (Malawi 3375).

### Food practices / convenience

In both countries participants described in detail how they accommodated (or tried to accommodate) SQ-LNS in their daily meal routines. Most of the women did this: *It is good for me, if I were to be taking it always I wouldn’t have had any problems* (Ghana 1218, 1^st^ interview). Typically women reported that they mixed SQ-LNS with a small amount of porridge or some other food in the morning as instructed. However, in Ghana it was rather common for the interviewees to say that it was difficult for them to take *Nkatepa* like this interviewee: *It is difficult for me…but I try my best to take all* (Ghana 1637, 1^st^ interview). In Malawi, women typically found consuming *Chiponde* easy as this quote illustrates: *But for me, I just cook porridge then I put in the porridge and eat* (Malawi 3365, 1^st^ interview).

In Malawi, women described a very limited selection of daily food alternatives besides *nsima ndi ndiwo*, stiff maize porridge with sauce and porridge, *phala*. There were only a few who reported spreading *Chiponde* on bread or scones and a few mixing it in tea. In Ghana, women narrated more dish options they experimented mixing *Nkatepa* with (porridge, soups, bread, tea, *banku*, stews). Some women in Ghana said that when the food they liked mixing the *Nkatepa* with was not available, they skipped taking it: *I used it to take oats or rice water or porridge, it is good for me, but if I am to use it to eat soupy dishes, I am unable to eat it* (Ghana 1314, 1^st^ interview). There were also some other non-food related reasons reported as a cause for skipping*: I went to the hospital yesterday and I was given injection … as for that I don’t take it [SQ-LNS] together with the injection* (Ghana 1704, 1^st^ interview).

Later on in the trial, many participants reported that they liked eating SQ-LNS straight from the sachet. Also if they did not cook porridge or other suitable food, they consumed SQ-LNS as a snack. Additionally, in Malawi, a few women said that if there was no other food, they would eat *Chiponde* from the sachet: *When I can’t find any food then I just take the Chiponde and start eating* (Malawi 8076, 1^st^ interview). In Ghana, there were no such accounts.

### Needs perception and benefits

In Ghana, women were routinely given iron, vitamin and mineral supplements by health personnel at the pre- and antenatal clinics; in addition a few mentioned buying nutrient supplements from pharmacy shops, and some bought herbal medications from local herbalists. In Malawi, women mainly talked about being used to receiving “pills from the hospital” (iron and folic acid tablets and malaria prophylaxis), and a few used medications from local healers to make birthing easier or to prevent some illnesses of the newborn. In Ghana, women typically referred to *Nkatepa* as “blood tonic mixed with groundnut paste”. Similarly in Malawi, women said “they explained to me that there is medicine in *Chiponde*, to increase blood in the body”. Also, in both countries, women expressed ideas about maternal diet having an impact on the health and nutrition of both the mother and the infant. Therefore, the expectation for a supplement like SQ-LNS was that it should replace the usual pregnancy medications, make the mother and the infant healthy and strong, and help the baby to develop. However, some participants said SQ-LNS did not have any health promoting impact. Only a few of the women expressed the need to take a supplement to promote child growth; according to the women who did not see a need to take a supplement, children grew normally, whether the mother took supplements or not.

In addition to SQ-LNS, some women felt they needed to take other supplements especially if they did not eat SQ-LNS every day like this woman in Ghana: *I am not able to take it well because it is not sweet for me, so once in a while I mix it with Koko [porridge], so as I have not been taking it, my blood is not much....that is why I went to the hospital to buy this medicine, Pregnant Care* (Ghana 1637, 1^st^ interview). Pregnant Care contains iron, folic acid, zinc, copper, manganese, magnesium, potassium; and vitamins B6, B12, C, and K.

Often women compared SQ-LNS with their earlier experiences. Some considered it to be better for the health of the mother and the infant, but some still preferred pills and tablets. Also, when it came to side effects, some described various symptoms with tablets, and others with SQ-LNS. A typical way of comparing was*: When I had my first born, the way I use this medicine and the way I used the B complex and others, I can see that this one is good for me* (Ghana 1156, 1^st^ interview).

In both Ghana and Malawi, several women reported positive effects on their skin: *But also my body changed, it was returning to youth on the skin, it was smooth.* (Malawi 4026, 2^nd^ interview) *When I am taking it, I notice that there are no rashes on my body* (Ghana 1218, 2^nd^ interview).

### Social environment

In Ghana, the participants reported that their husbands and family members learned about *Nkatepa* either through the study team or by the participants themselves informing them. None of the women whom we interviewed reported seeking approval from their husbands before enrolling in the study; they just informed their husbands and family members about the study, like this woman described: *I told him that this is some type of medicine we are eating so now, if I go to the hospital they no longer give me pills, so they said the pills they will give us they have passed it through this one, so we should eat it will give us strength and he said ok* (Ghana 1613, 1^st^ interview).

In Malawi, women typically thought that they should get permission from their husbands to enroll in the study and start taking *Chiponde* as this woman expressed: *He allowed me that you can join* (Malawi 8255, 1^st^ interview). Husbands’ reactions were usually positive especially if the women discussed the issue before starting to consume *Chiponde*. Some husbands who returned home after working in South Africa or bigger towns in Malawi told their wives to drop-out of the study. In some cases, the husbands and other relatives also wanted to taste SQ-LNS.

In Ghana, very few of the participants reported sharing the *Nkatepa*. Those who shared did so because their family or friends wanted to find out what the *Nkatepa* was like. They also did not report that they shared it more than once. In Malawi, women frequently mentioned sharing. Participants reiterated that the nurses said *Chiponde* was meant only for the study mothers but there was a lot of social pressure for sharing. They talked about hiding *Chiponde* sachets in the house and how every now and then sachets went missing. Family structure in the study area in Malawi is mostly matrilineal and sisters and their mother live close to each other. Typically, participants reported sharing *Chiponde* with their children, their sister’s children and with their sisters and mothers. Selling and bartering were reported very infrequently in both countries.

## Discussion

In this study we examined the consumption of SQ-LNS among two cohorts of pregnant and lactating women in Ghana and Malawi. We based the analysis of adherence on weekly/bi-weekly survey data. The mean adherence to SQ-LNS was high (>70 %) but varied across individuals from almost full adherence to almost complete non-adherence. The mean adherence was lower in Ghana than in Malawi and there was a decreasing trend in acceptability over time in Ghana and an increasing trend in Malawi. In-depth interviews provided a range of narratives of acceptability and adherence.

The strengths of our study are the longitudinal mixed methods approach used in data collection, the assessment of both adherence and acceptability, and coding and analysis by two independent researchers who understood the local languages. Some limitations of the study are that we did not observe participants, there was a high level of interaction with study staff, and adherence was self-reported, thus making social desirability bias in reporting possible. Additionally, we cannot say that acceptability would always lead to adherence.

There is a paucity of previous literature on SQ-LNS interventions among pregnant and lactating women. Adherence has been reported in only two previous studies. In a 2 weeks study in Ghana most of the pregnant and lactating women reported consuming the supplements as instructed [3]. In Bangladesh, 64 % of the pregnant trial participants reported using SQ-LNS daily or almost daily from the 28^th^ weeks of gestation through the first 6 months of lactation [18, 24]. Additionally, adherence to a similar but larger quantity LNS has been reported to be high: in a 28 weeks study in Malawi among HIV positive lactating women, daily adherence to supplement (70 g/d) was 92.4 % [20] and in Burkina Faso, daily adherence to supplement (72 g/d) was 75.4 % [19]. Self-reported adherence in our trials in Ghana and in Malawi is consistent with these previous studies.

In our trials, participants in the in-depth interviews gave reasons for adherence to SQ-LNS. Many of them talked of expected benefits for themselves and their infants in relation to SQ-LNS. This is in line with the health belief model focusing on an individual’s likelihood to adopt a certain behavior that is relevant to a specific condition or disease [30]. Another possible reason for adherence was that some women believed that the medicines they usually received during pregnancy had been mixed into SQ-LNS. Similarly, in Guatemala the use of ready-to-use supplementary foods (RUSF) increased proportional to the product’s perception as a medicine [12]. Among Bangladeshi pregnant and lactating women, the most common reasons for high adherence were the benefits they perceived, either personally, for their infant, or in general [18]. Participants in our trials also emphasized that the product was recommended by health care personnel as a reason to follow the instructions on how to take the SQ-LNS. Despite this, sharing was a major issue related to adherence, albeit with more women in Malawi than in Ghana reporting sharing the SQ-LNS with family and friends. In the Bangladesh study, 18 % of women receiving SQ-LNS reported sharing some of the supplements [18].

The mean self-reported overall adherence to supplement consumption was lower in Ghana than in Malawi; over time, the mean adherence increased in Malawi but decreased in Ghana for all supplement types. A study evaluating the factors associated with variation in compliance to iron supplementation over time among Peruvian women reported a median adherence of 79 % (interquartile range 65–89 %) over pregnancy [32]. In the Peruvian study, women with low initial compliance achieved high compliance by the end of pregnancy, similar to what was observed among Malawian women but in contrast to Ghanaian women. This could be partly attributed to the fact that in our study, Ghanaian women were more critical of the organoleptic properties of SQ-LNS and reported that it was difficult to take or they did not like it, whereas Malawian women were more positive and liked the supplement. In Peru, positive health reports were associated with greater compliance, and negative reports were associated with lower compliance [32].

In our trials, women were given a minimal set of instructions on how to take SQ-LNS (daily intake, not sharing, and mixing with a small amount of food) with consistent and reliable supply of the supplement at home. Other studies have shown that guaranteed supplement supply coupled with minimum, consistent and easily understandable information are key to effective pre- and postnatal supplementation programmes [6, 16].

Rather little is known about acceptability of SQ-LNS among this group of consumers: in the 2 weeks study in Ghana, SQ-LNS were well accepted [3] and in Bangladesh, the overall acceptability score for SQ-LNS was high [18]. In all of these studies as well as in our study, sensory/organoleptic (taste, mouth feel, after taste and smell) attributes were central to acceptability. Similar issues have been reported with other types of supplements. In a trial among pregnant women in Mexico, micronutrient powders and tablets were preferred over fortified food (*Nutrivida*) because participants disliked the smell, taste and texture of the food supplement [31]. Bangladeshi women suggested that specific preparation techniques (taking supplements with or without certain foods) could make consumption of SQ-LNS easier but the smell could still be a challenge [18].

In our trials, acceptability differed by country and there might be several reasons for this: there were more women in Malawi that perceived their households as food insecure than in Ghana, and staple foods were different with more variation in Ghana [4, 9]. It is therefore plausible that women in Ghana were more critical about the felt qualities of supplements, and Malawian women more willingly consume supplements given to them.

In our trials, some participants experienced nausea after consuming SQ-LNS whilst pregnant but nausea symptoms subsided in late pregnancy. Very few participants reported SQ-LNS-related nausea or vomiting during lactation. Among Bangladeshi pregnant and lactating women receiving SQ-LNS, low-adherers reported the ‘bad smell of the supplement’, and ‘feeling nausea and vomiting’ as some of the reasons for their consumption patterns [18]. Similar results have been reported with for example iron supplementation: pregnant women may be reluctant to take iron supplements because of the gastrointestinal discomfort [15, 23].

A few participants reported sharing SQ-LNS in Ghana to satisfy the curiosity of family and friends who wanted to find about the contents of the SQ-LNS sachets. Sharing was more frequent in Malawi, and can be attributed to social pressure and matrilineal family structure. However, because according to the in-depth interviews the children received only a small portion of mother’s supplement, it is not likely to be harmful to them.

## Conclusions

Self-reported sustained adherence to consume SQ-LNS daily was high in both sites but lower in Ghana than in Malawi. In Ghana adherence decreased over time whereas in Malawi adherence increased. Acceptability and adherence appeared interlinked, complex and context related. Achieving sustained consumption of SQ-LNS may require tailoring user instructions, health promotion messages and possibly the supplementary products by preferences of the intended consumers.

## Additional file

Additional file 1: Figure S1a. Participant flow in Ghana, CONSORT recommended format. **Figure S1b.** Participant flow in Malawi, CONSORT recommended format. (ZIP 10 kb)

## Abbreviations

IFA: Iron and folic acid tablets
MMN: Multiple micronutrient tablets
SD: Standard deviation
SQ-LNS: Small quantity lipid-based nutrient supplement
