# Supplementary figures and images for: A mixed method study exploring adherence to and acceptability of small quantity lipid-based nutrient supplements (SQ-LNS) among pregnant and lactating women in Ghana and Malawi
Source: BMC Pregnancy Childbirth. 2016 Aug 30;16(1):253. doi: 10.1186/s12884-016-1039-0 (PMC5004276; doi:10.1186/s12884-016-1039-0)

*Supplemental Figure 1b: Participant flow in Malawi, CONSORT recommended format*

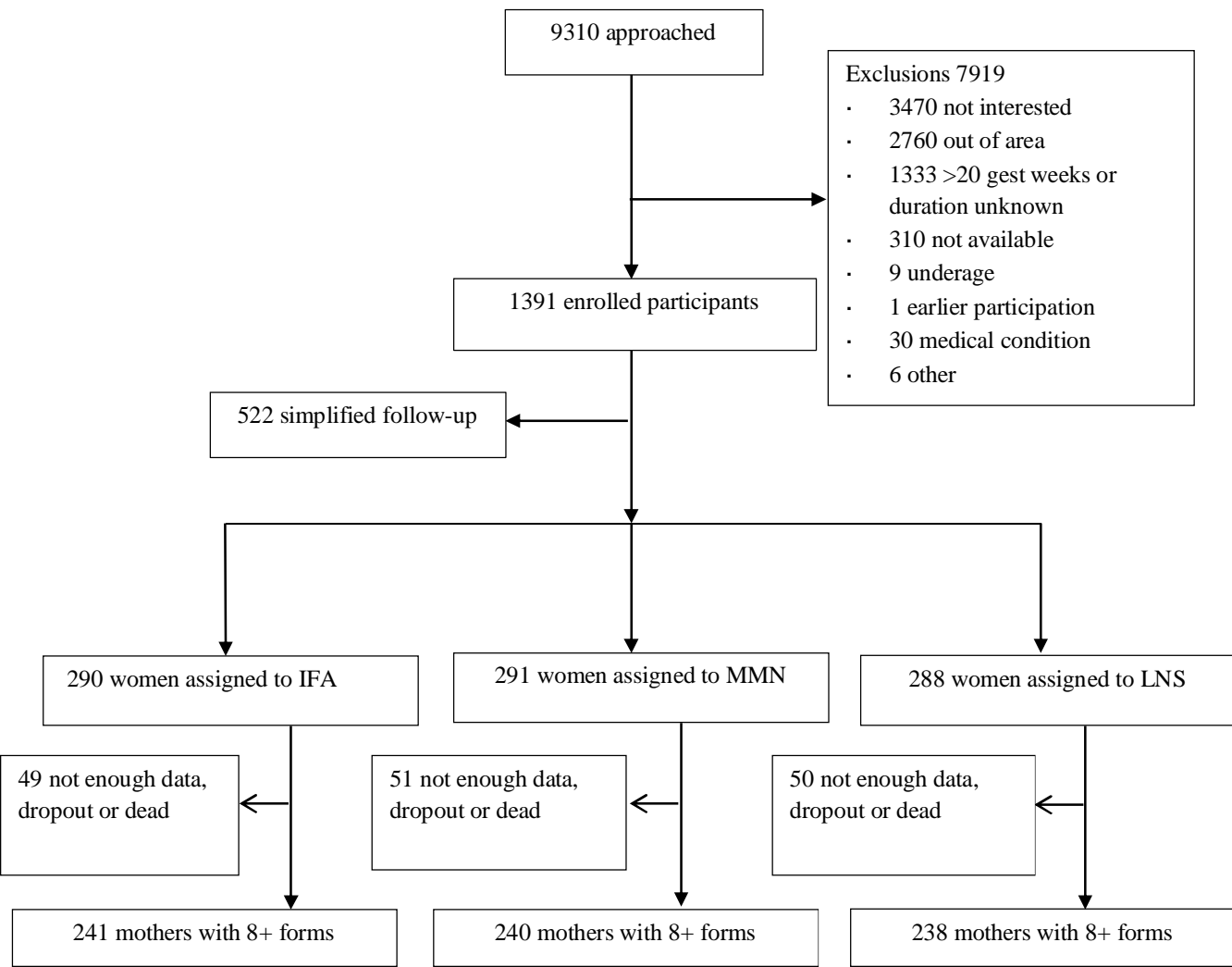

Supplement: Additional file 1: Figure S1a. — Participant flow in Ghana, CONSORT recommended format. Figure S1b. Participant flow in Malawi, CONSORT recommended format. (ZIP 10 kb) [file 12884_2016_1039_MOESM1_ESM.zip › Supplemental Figure/Supplemental Figure 1aR2.pdf]

*Supplemental Figure 1b: Participant flow in Ghana, CONSORT recommended format*

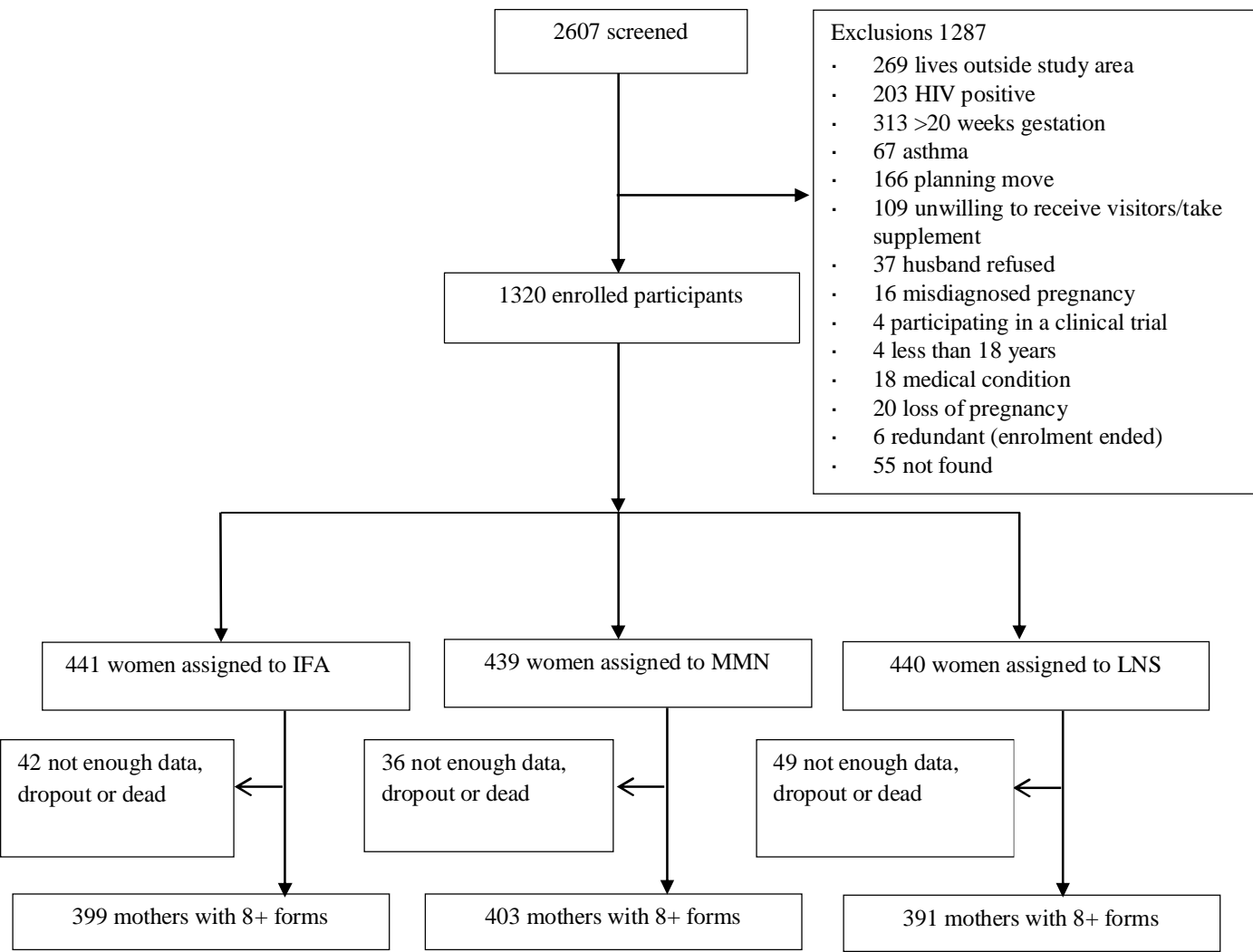

Supplement: Additional file 1: Figure S1a. — Participant flow in Ghana, CONSORT recommended format. Figure S1b. Participant flow in Malawi, CONSORT recommended format. (ZIP 10 kb) [file 12884_2016_1039_MOESM1_ESM.zip › Supplemental Figure/Supplemental Figure 1bR2.pdf]
